# DNAfusion: an R/Bioconductor package for increased sensitivity of detecting gene fusions in liquid biopsies

**DOI:** 10.1186/s12859-023-05259-3

**Published:** 2023-04-04

**Authors:** Christoffer Trier Maansson, Emma Roger Andersen, Maiken Parm Ulhoi, Peter Meldgaard, Boe Sandahl Sorensen

**Affiliations:** 1 Department of Clinical Biochemistry, Faculty of Health, Aarhus University Hospital, Palle Juul-Jensens Boulevard 69, 8200 Århus N, Denmark; 2 Department of Clinical Medicine, Aarhus University, Århus N, Denmark; 3 Department of Oncology, Aarhus University Hospital, Århus N, Denmark

**Keywords:** Gene fusion, EML4-ALK, R/Bioconductor, Non-small cell lung cancer, Next-generation sequencing, Variant call, Biomarker, Liquid biopsies

## Abstract

**Background:**

EML4-ALK gene fusions are oncogenic drivers in non-small cell lung cancer (NSCLC), and liquid biopsies containing *EML4-ALK* fragments can be used to study tumor dynamics using next-generation sequencing (NGS). However, the sensitivity of *EML4-ALK* detection varies between pipelines and analysis tools.

**Results:**

We developed an R/Bioconductor package, DNAfusion, which can be applied to BAM files generated by commercially available NGS pipelines, such as AVENIO. Forty-eight blood samples from a training cohort consisting of 41 stage IV *EML4-ALK*-positive NSCLC patients and seven healthy controls were used to develop DNAfusion. DNAfusion detected *EML4-ALK* in significantly more samples (sensitivity = 61.0%) compared to AVENIO (sensitivity = 36.6%). The newly identified *EML4-ALK*-positive patients were verified using droplet digital PCR. DNAfusion was subsequently validated in a blinded validation cohort comprising 24 *EML4-ALK*-positive and 24 *EML4-ALK*-negative stage IV NSCLC patients. DNAfusion detected significantly more *EML4-ALK* individuals in the validation cohort (sensitivity = 62.5%) compared to AVENIO (sensitivity = 29.2%). DNAfusion demonstrated a specificity of 100% in both the training and validation cohorts.

**Conclusion:**

Here we present DNAfusion, which increases the sensitivity of *EML4-ALK* detection in liquid biopsies and can be implemented downstream of commercially available NGS pipelines. The simplistic method of operating the R package makes it easy to implement in the clinical setting, enabling wider expansion of NGS-based diagnostics.

**Supplementary Information:**

The online version contains supplementary material available at 10.1186/s12859-023-05259-3.

## Background

Gene fusions are important oncogenic drivers in lung cancer, and the discovery of new fusions is ongoing [1]. For non-small cell lung cancer (NSCLC), this has led to the development of tyrosine kinase inhibitors (TKIs) specifically targeting the resulting fusion proteins, including ALK [2], ROS1 [3], RET [4], and NTRK [5]. Until now, these gene fusions have been identified in tissue biopsies [6], but the recent development of liquid biopsies offers the possibility to study tumor genetics in plasma samples [7]. In NSCLC, gene fusions involving *ALK* are the most comprehensively studied, and several TKIs targeting ALK-fusions are available for the treatment of patients with ALK positive NSCLC [8]. The most common *ALK* fusion partner is *EML4*, and *EML4-ALK* has been studied extensively in liquid biopsies. However, different next-generation sequencing (NGS) approaches and downstream software solutions have resulted in varying sensitivity for the detection of *EML4-ALK* in circulating tumor DNA (ctDNA). Sensitivity varies between 30 and 80% [9–18] depending on the choice of NGS and the bioinformatic pipeline. Gene fusions can be identified using commercially available hybridization capture-based methods, including AVENIO (Roche) [12, 19, 20], Guardant360 (GuardantHealth) [10, 16], and InVisionFirst (Inivata) [13]. Although these pipelines are simple to implement in the clinic and the output can be interpreted easily, they suffer from reduced sensitivity compared to results generated with, for example, Tophat2 [21] and FACTERA [22], which on the other hand require considerable bioinformatic knowledge to be implemented in routine diagnostics.

Here we present the R package, DNAfusion (available through Bioconductor), which can be implemented downstream of NGS pipelines to increase the sensitivity of *EML4-ALK* detection. DNAfusion is available across computer platforms through R. It is easy to install and use, making it applicable for clinical research and diagnostics. Here we compare the results of DNAfusion with the AVENIO output; however, it should be noted that BAM files generated with any hybridization capture-based paired-end sequencing can be used as input in DNAfusion.

## Results

### DNAfusion

**Fig. 1:**
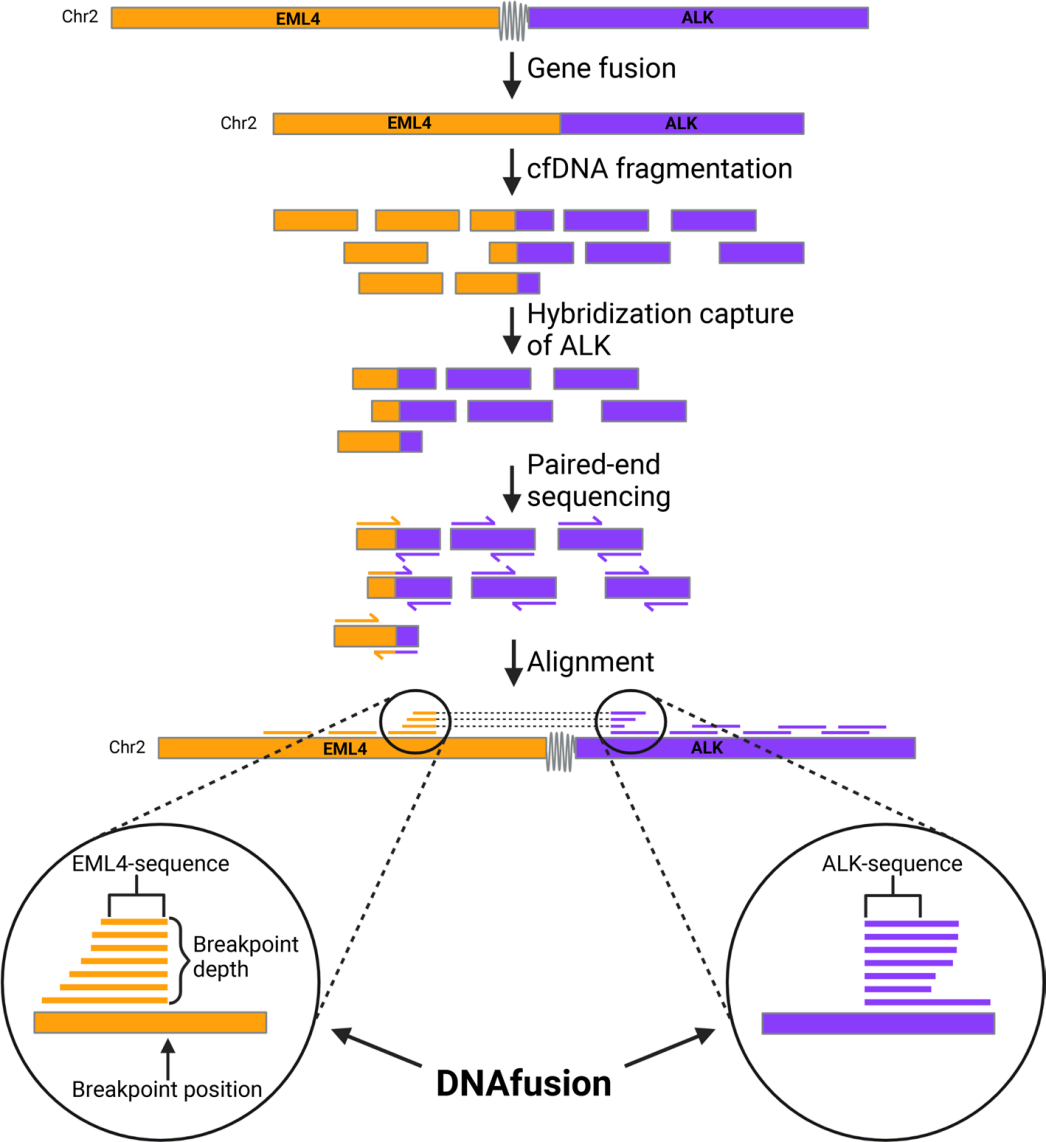
Identification of *EML4-ALK* variants using hybridization capture NGS and DNAfusion. *EML4* and *ALK* localized on chr2 can make a gene fusion. During cell-free DNA (cfDNA), release to the bloodstream the DNA is fragmented into approximately 165 bp fragments. The hybridization capture of *ALK* fragments will isolate *ALK* fragments but also *EML4-ALK* fusion fragments. Paired-end sequencing will generate reads aligning to *EML4* and *ALK* with reads spanning the fusion point becoming soft clipped. DNAfusion detects *EML4* reads with a mate in *ALK* and soft-clipped reads spanning the fusion breakpoint are identified. The bases leading up to the *EML4* breakpoint and following the *ALK* breakpoint are determined. Created with BioRender.com

DNAfusion utilizes paired-end sequencing to discover *EML4-ALK* fragments in liquid biopsies, as described in Fig. 1. Following *EML4-ALK* gene fusion, cfDNA fragmentation results in gene fragments from *EML4* and *ALK*, as well as some fragments spanning the fusion breakpoint. Hybridization capture of *ALK* fragments (e.g., in the AVENIO pipeline) also captures fragments with *EML4* fused to the *ALK* gene. After paired-end sequencing, DNAfusion identifies *EML4-ALK* fusion fragments by isolating soft-clipped *EML4* reads with a mate position in *ALK* (for methodological details se Methods). DNAfusion can characterize the gene fusion by identifying the breakpoint position and the DNA sequences around the breakpoint in both *EML4* and *ALK*. Furthermore, DNAfusion determines the read depth at the breakpoint in *EML4.* This serves as a surrogate for the ctDNA load in the blood sample, which in turn reflects the tumor burden [23–25]. Monitoring the read depth at the *EML4* breakpoint provides the possibility to monitor treatment efficacy because a reduction in ctDNA levels early during therapy is correlated with positive treatment response, including progression-free survival and overall survival [26]. Examples of two *EML4-ALK*-positive BAM files identified with DNAfusion are displayed in Additional file 1: Fig. S1.

### Comparing detection of *ALK* fusions with the AVENIO software and DNAfusion

**Fig. 2:**
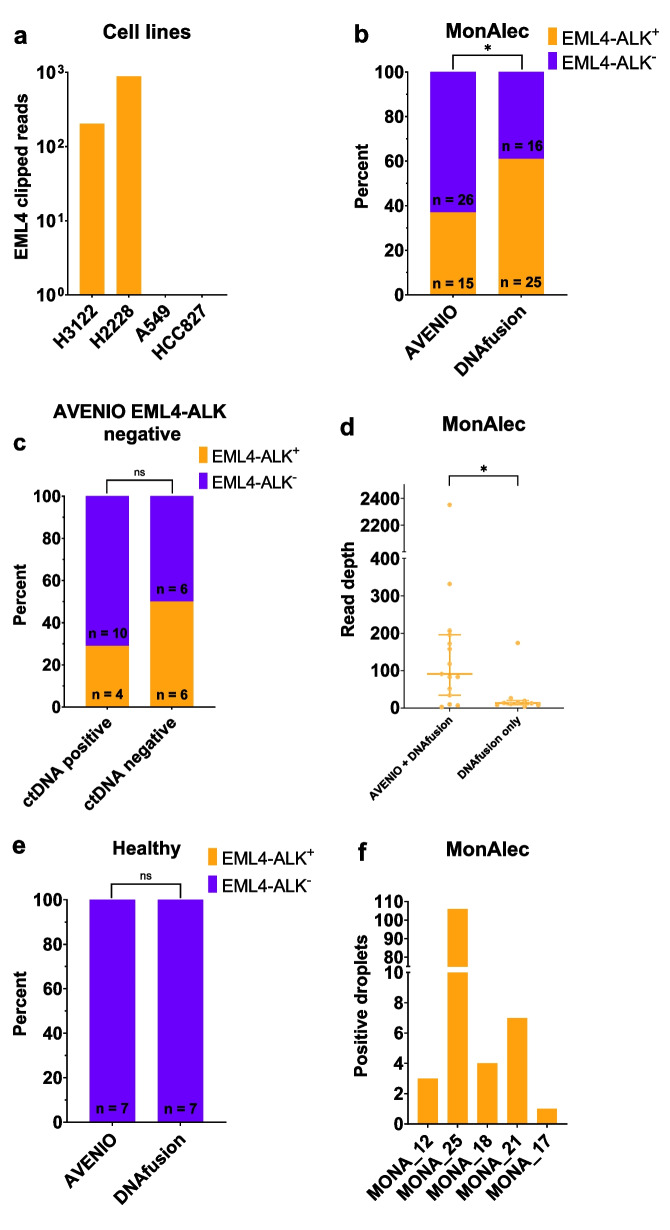
Comparing DNAfusion with AVENIO in the training cohort. **a** Number of EML4 clipped reads determined with DNAfusion in cell lines. **b** Number of patients with detectable and undetectable *EML4-ALK* in liquid biopsies, determined with either AVENIO or DNAfusion (*n* = 41). **c** Number of patients who are *EML4-ALK* positive or negative determined with DNAfusion in patients who are *EML4-ALK* negative with AVENIO. Patients are grouped according to their ctDNA status apart from *EML4-ALK* as determined with AVENIO. **d** The *EML4* breakpoint read depth in patients positive for *EML4-ALK* in both AVENIO and DNAfusion (*n* = 15) or only DNAfusion (*n* = 10). Median and IQR are indicated. **e** Number of healthy individuals with detectable or undetectable *EML4-ALK* in plasma, determined with either AVENIO or DNAfusion (*n* = 7). **f** Number of positive droplets for *EML4-ALK* in patients identified as *EML4-ALK* positive with DNAfusion but not with AVENIO. *P < 0.05

We created DNAfusion in order to increase the sensitivity of detecting *EML4-ALK* fragments, without affecting the specificity, in liquid biopsies following sequencing with the AVENIO pipeline. We therefore compared the outputs of the AVENIO Oncology Analysis software with the DNAfusion output (Fig. 2).

First, we evaluated the ability of DNAfusion to detect *EML4-ALK* in NSCLC cell lines. We tested the two *EML4-ALK*-positive cell lines, H3122 and H2228 [27], and the *EML4-ALK*-negative cell lines, A549 and HCC827. In Fig. 2a, the number of identified clipped *EML4* reads representing *EML4-ALK* reads is displayed for each cell line. Only *EML4-ALK* cell lines had detectable clipped reads. We then compared the AVENIO software with DNAfusion on the MonAlec cohort (Fig. 2b**,** Additional file 2: Table S1). DNAfusion identified significantly more *EML4-ALK*-positive patients compared to AVENIO (Fisher’s exact test: *P* = 0.046). The addition of DNAfusion increased the sensitivity from 36.6% (15/41) to 61.0% (25/41). All *EML4-ALK*-positive patients identified with AVENIO were also identified with DNAfusion (Additional file 2: Table S1). The AVENIO pipeline will identify mutations in 197 frequently mutated genes in NSCLC. We wanted to investigate whether the presence of additional mutations besides *EML4-ALK* fusions in the plasma (ctDNA positive) was associated with the increased DNAfusion sensitivity. We classified the *EML4-ALK*-negative patients determined with AVENIO (*n* = 26) as either ctDNA negative (*n* = 12) or ctDNA positive (*n* = 14) (Additional file 2: Table S2) and determined their *EML4-ALK* status with DNAfusion. As displayed in Fig. 2c, DNAfusion sensitivity is not dependent on the presence or absence of ctDNA. We tested the read depth at the *EML4* breakpoint for the patients identified as positive either with both AVENIO and DNAfusion or with DNAfusion only (Fig. 2d). Unsurprisingly, the read depth was significantly higher (Mann–Whitney test, *P* = 0.021) for the 15 patients identified with both AVENIO and DNAfusion compared to DNAfusion only (*n* = 10). This indicates that DNAfusion can detect *EML4-ALK* fusions with fewer *EML4-ALK* reads compared to AVENIO, although there is also an overlap between the read depths of the two groups. To address the specificity of DNAfusion we tested seven healthy controls and did not detect any *EML4-ALK* in any of the individuals (specificity = 100%, Fig. 2e). Of the 10 *EML4-ALK*-positive patients identified with DNAfusion but not with AVENIO, five patients had plasma available and were tested with droplet digital PCRC (ddPCR). Based on the output of DNAfusion identifying the *EML4* and *ALK* sequences surrounding the breakpoint, patient-specific primer and probe sets were designed (Additional file 2: Table S3, Additional file 1: Fig. S2). *EML4-ALK*-positive droplets were identified in all five plasma samples (Fig. 2f). It should be noted that only a single *EML4-ALK* droplet was detected in MONA_17, but three or more droplets were detected for the remaining four patients.

### Validation of DNAfusion

**Fig. 3:**
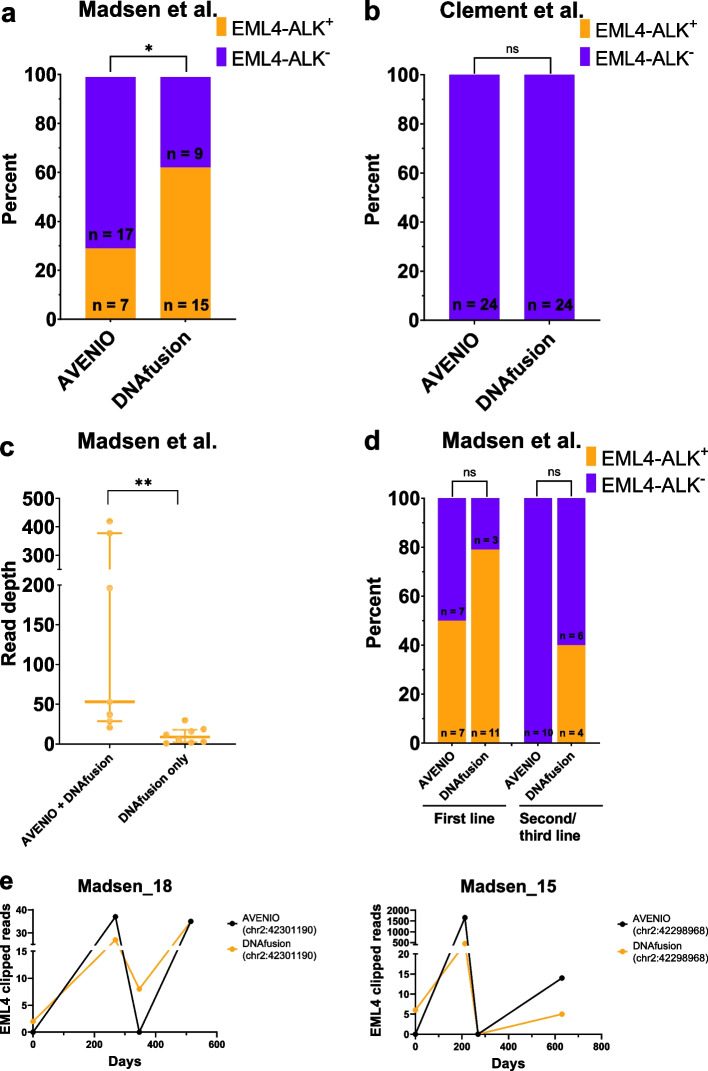
DNAfusion and AVENIO results of the validation cohort. **a** Number of patients with detectable and undetectable *EML4-ALK* in liquid biopsies from the Madsen cohort, determined with either AVENIO or DNAfusion (*n* = 24). **b** Number of patients with detectable and undetectable *EML4-ALK* in liquid biopsies from the Clement cohort, determined with either AVENIO or DNAfusion (*n* = 24). **c** The *EML4* breakpoint read depth in patients positive for *EML4-ALK* in both AVENIO and DNAfusion (*n* = 6) or only DNAfusion (*n* = 8). Median and IQR are indicated. **D** *EML4-ALK* positivity for AVENIO and DNAfusion for the Madsen cohort patients before the first line (*n* = 14) or second/third line (*n* = 10) ALK treatment. **e** Number of *EML4* clipped reads detected with either AVENIO or DNAfusion throughout the follow-up of Madsen_18 and Madsen_15. *P < 0.05; **P < 0.01

To verify the findings in Fig. 2, we combined NGS outputs from a previous study by Madsen et al. (*n* = 24) [28], of EML4-ALK positive patients, and NGS outputs from another study by Clement et al. (*n* = 24) [29], of EGFR-mutated but EML4-ALK-negative patients, to make a joint validation cohort (*n* = 48). The BAM files were blinded, meaning that the data interpreter did not know which of the two cohorts the plasma sample was from during the DNAfusion analysis. The results of the validation cohort are displayed in Fig. 3.

Similar to the results in Fig. 2b, Fig. 3a demonstrates that DNAfusion detected significantly more *EML4-ALK* patients (15/24, sensitivity = 62.5%) compared to AVENIO (7/24, sensitivity = 29.2%, Fisher’s exact test, *P* = 0.042, Additional file 2: Table S4). Additionally, analysis of the blinded negative controls of the Clement cohort demonstrated a specificity of 100% for both AVENIO and DNAfusion (Fig. 3b). Like the results of the MonAlec cohort, the *EML4-ALK*-positive patients only detected with DNAfusion had a lower read depth than the patients identified with both AVENIO and DNAfusion (Fig. 3c). All of the patients in the MonAlec cohort had their blood samples drawn before initiation of ALK TKI therapy, but 10/24 patients in the Madsen cohort had initiated ALK TKI therapy before NGS analysis. None of these patients were identified as *EML4-ALK* positive with AVENIO analysis; however, DNAfusion could detect *EML4-ALK* in four of these patients, which demonstrates the ability of DNAfusion to detect minimal residual ctDNA during TKI therapy. Furthermore, DNAfusion also detected more *EML4-ALK*-positive patients in the treatment-naïve cohort (Fig. 3d). Two patients from the Madsen cohort (patient Madsen_18 and Madsen_15) were identified as *EML4-ALK*-negative with AVENIO at the time of the first NGS, whereas an *EML4-ALK* fusion was detected in a later blood sample. Importantly, the breakpoint position in *EML4* detected in the baseline blood sample with DNAfusion, was identical to the breakpoint position identified with AVENIO in a later blood sample (Fig. 3e). This demonstrates how DNAfusion can detect *EML4-ALK* at lower amounts of ctDNA which is crucial for the detection of disease relapse during treatment. In addition, the third blood sample analyzed for Madsen_18 demonstrates a complete clearance of the *EML4-ALK* fusion with AVENIO; however, DNAfusion detects residual *EML4-ALK* mutant molecules.

## Discussion

The AVENIO pipeline is a commercially available NGS workflow where the output is easy to interpret for researchers without bioinformatic knowledge. However, we found that AVENIO has reduced sensitivity for the detection *EML4-ALK* in liquid biopsies [12, 28], compared to other more refined bioinformatic tools [10, 13, 15, 17]. We therefore developed DNAfusion, an easy-to-use and simple to implement tool, to be used downstream of the AVENIO pipeline that could increase the sensitivity. Through R, DNAfusion works fast (Additional file 1: Fig. S3) and across computer platforms, making it suitable for flexible implementation in the clinical setting.

A limitation of the presented study is that the results are solely based on NGS with the AVENIO pipeline, however DNAfusion will work with any capture-based NGS of cfDNA. Furthermore, the study is limited by the small *EML4-ALK*-positive (*n* = 65) and *EML4-ALK*-negative (*n* = 31) cohort sizes. The limited cohort size could result in over- or underestimation of the sensitivity and specificity for DNAfusion compared to AVENIO. Although DNAfusion identifies *EML4-ALK* in more plasma samples than the AVENIO pipeline, these findings need to be validated in a larger cohort using different NGS pipelines.

Applying AVENIO and DNAfusion provides the opportunity to monitor the ctDNA dynamics of both gene fusions as well as other somatic mutations. At the time of writing this paper DNAfusion has been developed to target *EML4-ALK* fusions, but we plan to expand the number of *ALK* fusion partners in the future. Furthermore, we plan to develop a similar approach toward other gene fusions, such as ROS1 [3], RET [4], and NTRK [5] in NSCLC, but also gene fusions identified in other solid cancers, such as TMPRSS2-ERG in prostate cancer [30], EVT6-NTRK3 in secretory breast carcinoma [31] etc.

In Fig. 3e, it is demonstrated how DNAfusion can detect *EML4-ALK* molecules earlier than AVENIO. The fact that AVENIO detects the same *EML4-ALK* fusion breakpoint in a later blood sample from the same patient demonstrates how the *EML4-ALK* fusion detected with DNAfusion in an earlier blood sample is not a false positive result based on a low number of reads. This result clearly shows how DNAfusion can detect *EML4-ALK* fusions in a blood sample with a lower ctDNA load than AVENIO. Furthermore, the third NGS of Madsen_18 analyzed with AVENIO misclassifies the patient as a clearer of the *EML4-ALK* fusion; however, re-analysis with DNAfusion detected residual *EML4-ALK* molecules classifying the patient as a non-clearer. This is clinically relevant information given that ctDNA clearance predicts the outcome during TKI treatment [18, 32, 33] and illustrates how DNAfusion is more sensitive in classifying patients as clearers or non-clearers.

## Conclusion

In this study, we present DNAfusion, an R/Bioconductor package for detection of *EML4-ALK* fusions with NGS. In both the training cohort and the validation cohort, DNAfusion displayed higher sensitivity than the AVENIO algorithm regarding the detection of *EML4-ALK* molecules and maintained a specificity of 100%. The straightforward implementation of DNAfusion can help expand the utility of NGS-based diagnostics in the clinic and guide treatment decisions.

## Methods

### Patient cohorts

Eighty-nine stage IV NSCLC patients and seven healthy controls were included in this study (Additional file 1: Fig. S4). Sixty-five patients were EML4-ALK positive, of which 41 are part of the ongoing prospective MonAlec study (NCT04708639) [34] and 24 were part of a previous EML4-ALK cohort published by Madsen et al. [28] (Called the Madsen cohort, Additional file 2: Table S4). *ALK* rearrangements were tested as part of the routine diagnostics using fluorescence in situ hybridization (FISH) or NGS analysis of tissue samples. The seven healthy controls were used as *EML4-ALK* negative controls alongside 24 NSCLC patients from the EGFR positive cohort (NCT02284633) published by Clement et al. [29] (called the Clement cohort).

### Blood samples

Blood samples were collected in 10 mL K2EDTA (BD vacutainer, Becton, Dickinson and Company, Franklin Lakes, NJ, USA) tubes, however, for the MonAlec study the blood was drawn in 10 mL Cell-free DNA BCT tubes (Streck Corporate, La Vista, NE, USA). For the Clement and MonAlec cohorts, all baseline blood samples were drawn before treatment initiation. For the Madsen cohort, 14/24 patients were TKI treatment naïve, and 10 had received prior TKI therapy at the baseline blood sample collection [28]. The plasma was isolated within 2 h of extraction by centrifugation at 1900*g* for 10 min and subsequently stored at − 80 °C. When thawed, the plasma was centrifuged again at 16,000*g* for 10 min.

### Cell lines

NSCLC cell lines with EML4-ALK [27], H2228 (CRL-5935, ATCC, LCG standards, Wesel, Germany), and H3122 (Adi F. Gazdar, UT Southwestern, Dallas, TX, USA), or without EML4-ALK, HCC827 (CRL-2868, ATCC, LCG standards, Wesel, Germany), and A549 (CCL-185, ATCC, LCG standards, Wesel, Germany), were grown in an RPMI medium with 10% fetal calf serum and 1% penicillin–streptomycin solution. All cells were cultivated in 5% CO_2_ at 37 °C. Approximately 2.0 × 10^6^ cells were lysed in 100 µL lysis buffer (Tris–HCl 50 mM, EDTA 10 mM, NP-40 1%, SDS 1%) and physically fragmented by sonication to an average fragment length of 200–500 bp. The DNA was treated with 20 µg Proteinase K and purified using the NucleoSpin Gen and PCR Clean-up kit (Macherey–Nagel, Dueren, Germany). The purified DNA was kept at − 20 °C until applied to NGS.

### Cancer personalized profiling by deep sequencing (CAPP-seq)

Cell-free DNA (cfDNA) was isolated from 2.0 to 4.0 mL plasma using the AVENIO cfDNA Isolation Kit (Roche Sequencing Solutions, Mannheim, Germany). cfDNA concentrations were estimated with the Qubit dsDNA HS Assay Kit (Thermo Fisher Scientific, Waltham, MA, USA), and the fragment lengths were analyzed using Agilent 2100 Bioanalyzer (Agilent Technologies, Santa Clara, CA, USA). NGS libraries were prepared with the AVENIO ctDNA Library Prep Kit (Roche Sequencing Solutions, Mannheim, Germany) and AVENIO ctDNA Surveillance Panel (Roche Sequencing Solutions, Mannheim, Germany), covering 197 genes, or the AVENIO ctDNA Expanded panel (Roche Sequencing Solutions, Mannheim, Germany) for the Madsen cohort, covering 77 genes. The gene fragments were sequenced using 100 paired-end sequencing on NextSeq 500 (Illumina, San Diego, CA, USA). The BAM files and the AVENIO mutation call were made using the AVENIO Oncology Analysis software.

### Droplet digital PCR (ddPCR)

Droplet digital PCR (ddPCR) experiments were run in duplicates using the QX200 AutoDG Droplet Digital PCR System (Bio-Rad, Hercules, CA, USA). Each well containing 11 µL of ddPCR Supermix for Probes (no UTP), 1 µL of forward and reverse primer (20 µM), 1 µL of fluorescent probe (1 µM), and 8 µL of DNA sample to a total volume of 22 µL. The details of primers and probes are available in Additional file 2: Table S3. Droplets were generated using the QX200 AutoDG (Bio-Rad, Hercules, CA, USA), and the PCR was performed using a GeneAmp PCR System 9700 (Applied Biosystems, Waltham, MA, USA). Droplets were read using the QX200 Droplet Reader (Bio-Rad, Hercules, CA, USA), and the data were analyzed in QX Manager 1.2 Standard Edition.

### DNAfusion

The seven healthy controls and the MonAlec cohort (Additional file 2: Table S1) were used as a training set for DNAfusion. The Clement and Madsen (Additional file 2: Table S4) cohorts were combined and used as a separate validation cohort. The BAM files from the Madsen and Clement cohorts were blinded during analysis with DNAfusion enabling unbiased interpretation of the output. The position deduplicated BAM files were used as input for DNAfusion from all individuals. The *EML4_ALK_detection()* function was used to determine whether the plasma sample was *EML4-ALK* positive. *EML4_ALK_detection()* takes reads aligned to *hg38* or *hg19* and filters reads from *EML4* (chr2: 42169353-42332548 for *hg38*). Next, *EML4* reads with a paired read in *ALK* (chr2: 29192774-29921586 for *hg38*) is filtered. A threshold for the minimum number of *EML4-ALK* read pairs needed can be set (default = 2). Next, soft-clipped reads are identified as the reads with an “S” in the cigar string. The aligned part of these reads represents the DNA sequence (identified using *EML4_sequence()*) leading up to the *EML4*-breakpoint (identified using *break_position()*) whereas the soft-clipped part represents the DNA sequence (identified using *ALK_sequence()*) following the *ALK*-breakpoint. The minimum number of soft-clipped reads needed for the sample to be *EML4-ALK* positive, is set to 2 as default. If the *EML4_ALK_detection()* function identifies *EML4-ALK* it returns a GAlignments object containing *EML4* soft-clipped reads with a paired read in *ALK*. If *EML4-ALK* is not identified the GAlignments object is empty. DNAfusion was run in R version 4.2.1 and is available at https://bioconductor.org/packages/DNAfusion.

### Statistics

The ability for DNAfusion and the AVENIO software to detect *EML4-ALK* DNA fusions were compared using Fisher’s exact test. Differences in read depths were tested using a Mann–Whitney test. A two-sided *P* < 0.05 was considered significant. Statistical tests were performed in GraphPad Prism v. 9.3.1.

## Supplementary Information

**Additional file 1.** Supplementary figures 1–4.

**Additional file 2.** Supplementary tables 1–4.

## Data Availability

BAM files from CAPP-seq of cell lines have been deposited in the European Nucleotide Archive (ENA) at EMBL-EBI under accession number PRJEB56814 (https://www.ebi.ac.uk/ena/browser/view/PRJEB56814). DNAfusion is available at https://bioconductor.org/packages/DNAfusion and runs on Linux, MAC OS, and Microsoft-Windows. It requires R (version ≥ 4.2.0), which is available at https://cran.r-project.org and Bioconductor (version ≥ 3.16) available at https://bioconductor.org/install.

## Abbreviations

CAPP-seq: Cancer personalized profiling by deep sequencing
cfDNA: Cell-free DNA
ctDNA: Circulating tumor DNA
ddPCR: Droplet digital PCR
FISH: Fluorescence in situ hybridization
NGS: Next-generation sequencing
NSCLC: Non-small cell lung cancer
TKI: Tyrosine kinase inhibitor

## References

[1] Mertens F, Johansson B, Fioretos T, Mitelman F (2015). The emerging complexity of gene fusions in cancer. Nat Rev Cancer.

[2] Peters S, Camidge DR, Shaw AT, Gadgeel S, Ahn JS, Kim DW (2017). Alectinib versus crizotinib in untreated ALK-positive non-small-cell lung cancer. N Engl J Med.

[3] Drilon A, Siena S, Dziadziuszko R, Barlesi F, Krebs MG, Shaw AT (2020). Entrectinib in ROS1 fusion-positive non-small-cell lung cancer: integrated analysis of three phase 1–2 trials. Lancet Oncol.

[4] Gainor JF, Curigliano G, Kim DW, Lee DH, Besse B, Baik CS (2021). Pralsetinib for RET fusion-positive non-small-cell lung cancer (ARROW): a multi-cohort, open-label, phase 1/2 study. Lancet Oncol.

[5] Hong DS, DuBois SG, Kummar S, Farago AF, Albert CM, Rohrberg KS (2020). Larotrectinib in patients with TRK fusion-positive solid tumours: a pooled analysis of three phase 1/2 clinical trials. Lancet Oncol.

[6] Bruno R, Fontanini G (2020). Next generation sequencing for gene fusion analysis in lung cancer: a literature review. Diagnostics (Basel)..

[7] Rodríguez J, Avila J, Rolfo C, Ruíz-Patiño A, Russo A, Ricaurte L (2021). When tissue is an issue the liquid biopsy is nonissue: a review. Oncol Ther..

[8] Remon J, Pignataro D, Novello S, Passiglia F (2021). Current treatment and future challenges in ROS1- and ALK-rearranged advanced non-small cell lung cancer. Cancer Treat Rev.

[9] Liu L, Liu H, Shao D, Liu Z, Wang J, Deng Q (2018). Development and clinical validation of a circulating tumor DNA test for the identification of clinically actionable mutations in nonsmall cell lung cancer. Genes Chromosom Cancer.

[10] Supplee JG, Milan MSD, Lim LP, Potts KT, Sholl LM, Oxnard GR (2019). Sensitivity of next-generation sequencing assays detecting oncogenic fusions in plasma cell-free DNA. Lung Cancer.

[11] Horn L, Whisenant JG, Wakelee H, Reckamp KL, Qiao H, Leal TA (2019). Monitoring therapeutic response and resistance: analysis of circulating tumor DNA in patients with ALK+ lung cancer. J Thorac Oncol.

[12] Christopoulos P, Dietz S, Angeles AK, Rheinheimer S, Kazdal D, Volckmar AL (2021). Earlier extracranial progression and shorter survival in ALK-rearranged lung cancer with positive liquid rebiopsies. Transl Lung Cancer Res.

[13] Mezquita L, Swalduz A, Jovelet C, Ortiz-Cuaran S, Howarth K, Planchard D (2020). Clinical Relevance of an amplicon-based liquid biopsy for detecting ALK and ROS1 fusion and resistance mutations in patients with non-small-cell lung cancer. JCO Precis Oncol..

[14] Kunimasa K, Kato K, Imamura F, Kukita Y (2019). Quantitative detection of ALK fusion breakpoints in plasma cell-free DNA from patients with non-small cell lung cancer using PCR-based target sequencing with a tiling primer set and two-step mapping/alignment. PLoS ONE.

[15] Cui S, Zhang W, Xiong L, Pan F, Niu Y, Chu T (2017). Use of capture-based next-generation sequencing to detect ALK fusion in plasma cell-free DNA of patients with non-small-cell lung cancer. Oncotarget.

[16] Leighl NB, Page RD, Raymond VM, Daniel DB, Divers SG, Reckamp KL (2019). Clinical utility of comprehensive cell-free DNA analysis to identify genomic biomarkers in patients with newly diagnosed metastatic non-small cell lung cancer. Clin Cancer Res.

[17] Wang Y, Tian PW, Wang WY, Wang K, Zhang Z, Chen BJ (2016). Noninvasive genotyping and monitoring of anaplastic lymphoma kinase (ALK) rearranged non-small cell lung cancer by capture-based next-generation sequencing. Oncotarget.

[18] Kwon M, Ku BM, Olsen S, Park S, Lefterova M, Odegaard J (2022). Longitudinal monitoring by next-generation sequencing of plasma cell-free DNA in ALK rearranged NSCLC patients treated with ALK tyrosine kinase inhibitors. Cancer Med.

[19] Newman AM, Bratman SV, To J, Wynne JF, Eclov NC, Modlin LA (2014). An ultrasensitive method for quantitating circulating tumor DNA with broad patient coverage. Nat Med.

[20] Verma S, Moore MW, Ringler R, Ghosal A, Horvath K, Naef T (2020). Analytical performance evaluation of a commercial next generation sequencing liquid biopsy platform using plasma ctDNA, reference standards, and synthetic serial dilution samples derived from normal plasma. BMC Cancer.

[21] Kim D, Pertea G, Trapnell C, Pimentel H, Kelley R, Salzberg SL (2013). TopHat2: accurate alignment of transcriptomes in the presence of insertions, deletions and gene fusions. Genome Biol.

[22] Newman AM, Bratman SV, Stehr H, Lee LJ, Liu CL, Diehn M (2014). FACTERA: a practical method for the discovery of genomic rearrangements at breakpoint resolution. Bioinformatics.

[23] Diehl F, Li M, Dressman D, He Y, Shen D, Szabo S (2005). Detection and quantification of mutations in the plasma of patients with colorectal tumors. Proc Natl Acad Sci USA.

[24] Lam VK, Zhang J, Wu CC, Tran HT, Li L, Diao L (2021). Genotype-specific differences in circulating tumor DNA levels in advanced NSCLC. J Thorac Oncol.

[25] Winther-Larsen A, Demuth C, Fledelius J, Madsen AT, Hjorthaug K, Meldgaard P (2017). Correlation between circulating mutant DNA and metabolic tumour burden in advanced non-small cell lung cancer patients. Br J Cancer.

[26] Wang H, Zhou F, Qiao M, Li X, Zhao C, Cheng L (2021). The role of circulating tumor DNA in advanced non-small cell lung cancer patients treated with immune checkpoint inhibitors: a systematic review and meta-analysis. Front Oncol.

[27] Koivunen JP, Mermel C, Zejnullahu K, Murphy C, Lifshits E, Holmes AJ (2008). EML4-ALK fusion gene and efficacy of an ALK kinase inhibitor in lung cancer. Clin Cancer Res.

[28] Madsen AT, Winther-Larsen A, McCulloch T, Meldgaard P, Sorensen BS (2020). Genomic profiling of circulating tumor DNA predicts outcome and demonstrates tumor evolution in ALK-positive non-small cell lung cancer patients. Cancers (Basel)..

[29] Clement MS, Ebert EBF, Meldgaard P, Sorensen BS (2021). Co-occurring MET amplification predicts inferior clinical response to first line erlotinib in advanced stage EGFR-mutated NSCLC patients. Clin Lung Cancer.

[30] Wang Z, Wang Y, Zhang J, Hu Q, Zhi F, Zhang S (2017). Significance of the TMPRSS2:ERG gene fusion in prostate cancer. Mol Med Rep.

[31] Tognon C, Knezevich SR, Huntsman D, Roskelley CD, Melnyk N, Mathers JA (2002). Expression of the ETV6-NTRK3 gene fusion as a primary event in human secretory breast carcinoma. Cancer Cell.

[32] Ebert EBF, McCulloch T, Hansen KH, Linnet H, Sorensen B, Meldgaard P (2020). Clearing of circulating tumour DNA predicts clinical response to first line tyrosine kinase inhibitors in advanced epidermal growth factor receptor mutated non-small cell lung cancer. Lung Cancer.

[33] Boysen Fynboe Ebert E, McCulloch T, Holmskov-Hansen K, Linnet H, Sorensen B, Meldgaard P (2020). Clearing of circulating tumour DNA predicts clinical response to osimertinib in EGFR mutated lung cancer patients. Lung Cancer..

[34] Meldgaard P. Monitoring alectinib treatment by detection of ALK translocations in serial blood samples from non-small cell lung cancer patients (MonAlec), NCT04708639 https://clinicaltrials.gov2021 [Circulating tumor DNA can be used to monitor the treatment effect and identify developing resistance mutations during ALK directed TKI treatment.]. https://clinicaltrials.gov/ct2/show/NCT04708639

