# Supplementary material for: DNAfusion: an R/Bioconductor package for increased sensitivity of detecting gene fusions in liquid biopsies
Source: BMC Bioinformatics. 2023 Apr 4;24:131. doi: 10.1186/s12859-023-05259-3 (PMC10074784; doi:10.1186/s12859-023-05259-3)
Supplement: Supplementary file 1 — Additional file 1. Supplementary figures 1–4. [file 12859_2023_5259_MOESM1_ESM.docx]

# Additional file 1

## Supplementary figures


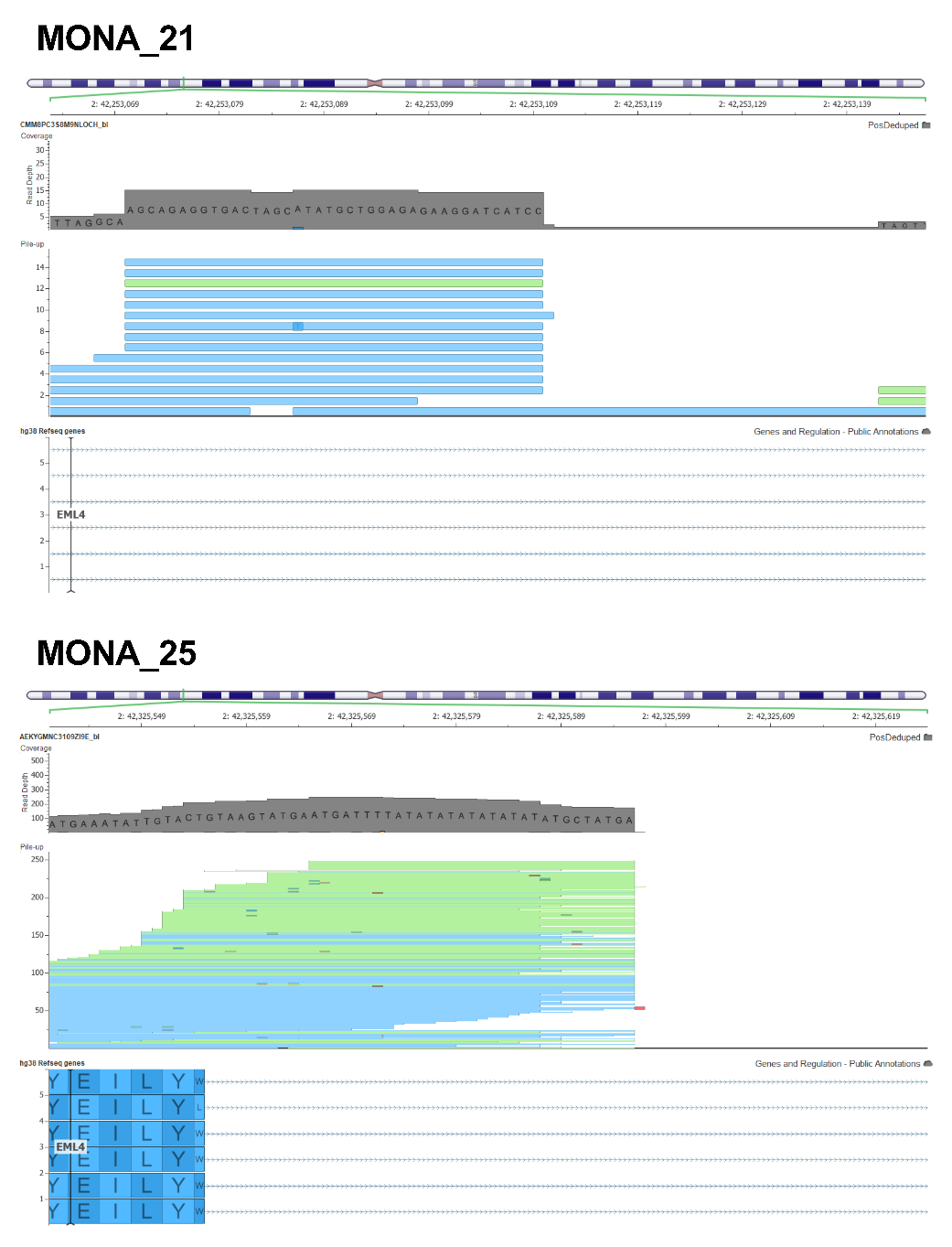


Fig S1. Examples of identified EML4 breakpoints with DNAfusion. DNAfusion and not AVENIO detected EML4-ALK in MONA_21 and MONA_25. The breakpoint positions in EML4 are chr2:42253109 and chr2:42325595 for MONA_21 and MONA_25 respectively. The reads surrounding the breakpoint are displayed using Golden Helix GenomeBrowse 3.0.0.


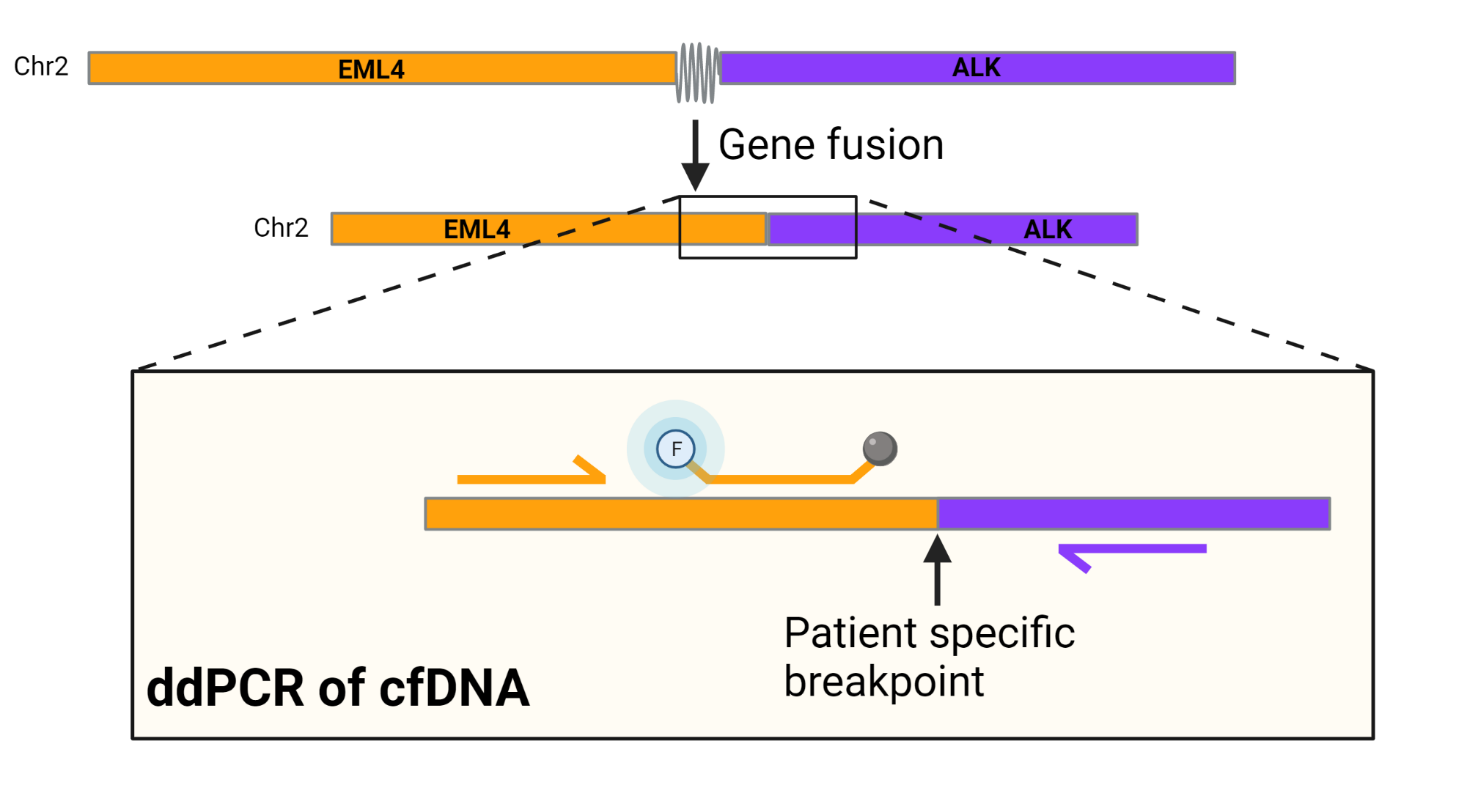


Fig. S2. Design of patient specific primers and probes. The patient specific breakpoint in *EML4* and *ALK* is identified with DNAfusion. Based on the sequences surrounding the breakpoint in *EML4* and in *ALK,* patient specific primer/probe sets were designed. The forward and reverse primers were split between *EML4* and *ALK* and the FAM-conjugated probe was either located in *EML4* or *ALK*. Created with BioRender.com.


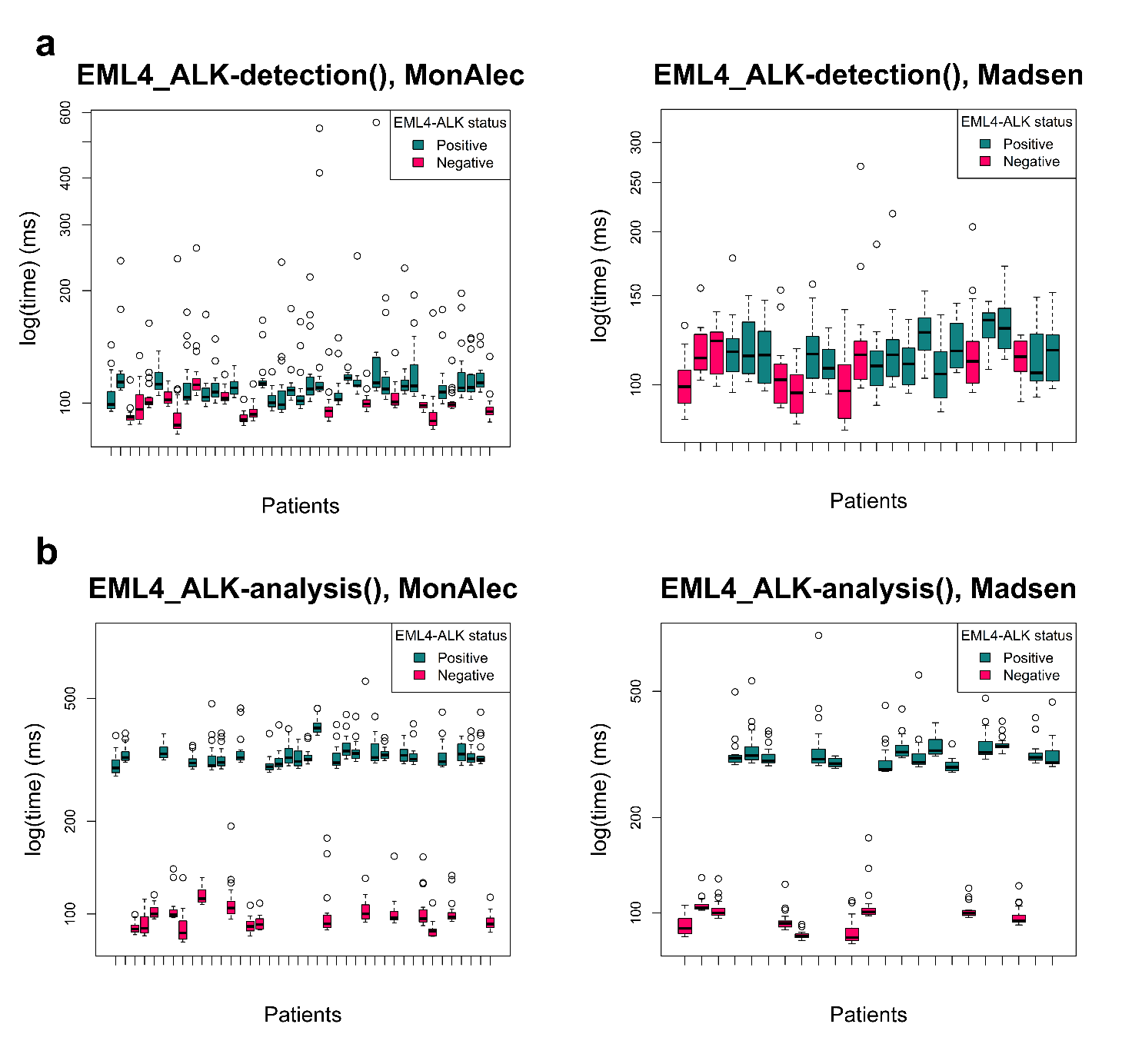


Fig. S3. Runtime of DNAfusion functions. Runtimes were estimated for the EML4_ALK-detection() **a** and EML4_ALK-analysis() **b** functions using microbenchmark v. 1.4.9 in R. The runtime was estimated for each patient in the MonAlec and the Madsen cohort 20 times. Patients identified as *EML4-ALK* positive are green, whereas *EML4-ALK* negative patients are red.


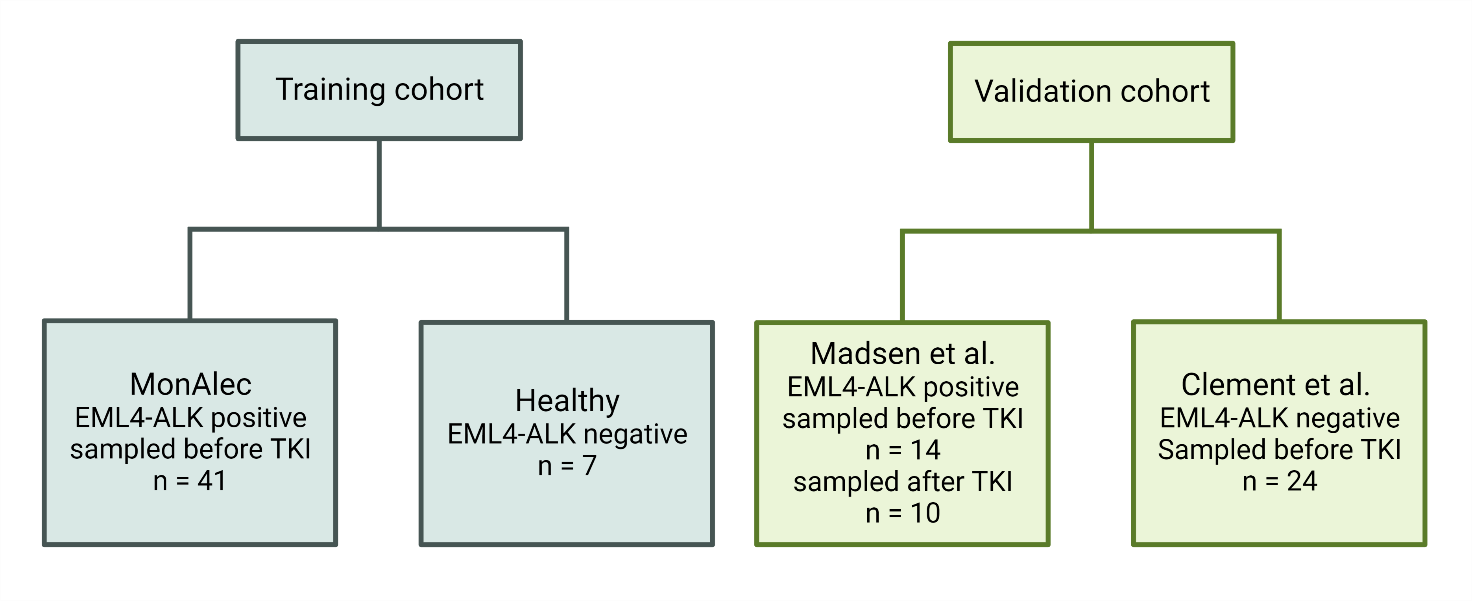


Fig. S4. Cohorts used in this study. The training cohort consists of blood samples from 7 healthy individuals and 41 samples from the MonAlec cohort. The validation cohort consists of 24 patients from the Clement cohort and 24 patients from the Madsen cohort. Created with BioRender.com.
