# Supplementary material for: DNAfusion: an R/Bioconductor package for increased sensitivity of detecting gene fusions in liquid biopsies
Source: BMC Bioinformatics. 2023 Apr 4;24:131. doi: 10.1186/s12859-023-05259-3 (PMC10074784; doi:10.1186/s12859-023-05259-3)
Supplement: Supplementary file 2 — Additional file 2. Supplementary tables 1–4. [file 12859_2023_5259_MOESM2_ESM.docx]

# Additional file 2

## Supplementary tables

Table S1. EML4-ALK status of the baseline samples in the MonAlec cohort. P: Positive, N: Negative.

| Patient ID | EML4-ALK status | |  | Reads spanning breakpoint | |  | EML4 breakpoint position |
| --- | --- | --- | --- | --- | --- | --- | --- |
|  | AVENIO | DNAfusion |  | AVENIO | DNAfusion |  |  |
| MONA_1 | P | P |  | 73 | 19 |  | 42299646 |
| MONA_2 | P | P |  | 122 | 49 |  | 42299776 |
| MONA_3 | N | N |  | - | - |  | - |
| MONA_4 | N | N |  | - | - |  | - |
| MONA_5 | N | N |  | - | - |  | - |
| MONA_6 | P | P |  | 371 | 282 |  | 42296960 |
| MONA_7 | N | N |  | - | - |  | - |
| MONA_8 | N | N |  | - | - |  | - |
| MONA_9 | P | P |  | 114 | 48 |  | 42299174 |
| MONA_10 | N | N |  | - | - |  | - |
| MONA_11 | P | P |  | 261 | 53 |  | 42274974 |
| MONA_12 | N | P |  | - | 6 |  | 42277430 |
| MONA_13 | N | N |  | - | - |  | - |
| MONA_14 | N | P |  | - | 6 |  | 42279176 |
| MONA_15 | N | N |  | - | - |  | - |
| MONA_16 | N | N |  | - | - |  | - |
| MONA_17 | N | P |  | - | 26 |  | 42265553 |
| MONA_18 | N | P |  | - | 14 |  | 42299795 |
| MONA_19 | P | P |  | 275 | 153 |  | 42297153 |
| MONA_20 | P | P |  | 431 | 87 |  | 42276703 |
| MONA_21 | N | P |  | - | 14 |  | 42253109 |
| MONA_22 | P | P |  | 213 | 105 |  | 42299182 |
| MONA_23 | P | P |  | 1424 | 1772 |  | 42270855 |
| MONA_24 | N | N |  | - | - |  | - |
| MONA_25 | N | P |  | - | 76 |  | 42325595 |
| MONA_26 | N | P |  | - | 8 |  | 42299165 |
| MONA_27 | P | P |  | 138 | 70 |  | 42295554 |
| MONA_28 | N | N |  | - | - |  | - |
| MONA_29 | P | P |  | 28 | 8 |  | 42300673 |
| MONA_30 | P | P |  | 583 | 228 |  | 42267333 |
| MONA_31 | N | N |  | - | - |  | - |
| MONA_32 | N | P |  | - | 3 |  | 42298211 |
| MONA_33 | P | P |  | 14 | 3 |  | 42297619 |
| MONA_34 | N | N |  | - | - |  | - |
| MONA_35 | N | N |  | - | - |  | - |
| MONA_36 | N | P |  | - | 14 |  | 42270900 |
| MONA_37 | N | N |  | - | - |  | - |
| MONA_38 | P | P |  | 12 | 11 |  | 42319394 |
| MONA_39 | N | P |  | - | 3 |  | 42300025 |
| MONA_40 | P | P |  | 31 | 6 |  | 42280879 |
| MONA_41 | N | N |  | - | - |  | - |

Table S2. ctDNA status besides *EML4-ALK* of MonAlec patients negative for *EML4-ALK* with AVENIO. P: Positive, N: Negative

| Patient ID | ctDNA status (Besides EML4-ALK) | Mutation (MAF%) |
| --- | --- | --- |
| MONA_3 | P | FBXW7-Ser86Leu (0.12%) |
| MONA_4 | P | BRAF-Val600Glu (2.67%) |
| MONA_5 | N |  |
| MONA_7 | N |  |
| MONA_8 | P | FGFR2-Ala499Val (0.60%) |
| MONA_10 | P | TP53-Arg273Cys (0.35%) |
| MONA_12 | P | FBXL7-Ala137Val (0.39%), TP53-Cys176Tyr (0.34%) |
| MONA_13 | P | TP53-Thr155Ile (13.63%) |
| MONA_14 | P | BRINP3-Arg719Cys (3.44%) |
| MONA_15 | N |  |
| MONA_16 | P | MET-Met1268Thr (0.08%) |
| MONA_17 | N |  |
| MONA_18 | N |  |
| MONA_21 | N |  |
| MONA_24 | N |  |
| MONA_25 | P | TP53-Met246Val (1.32%) |
| MONA_26 | N |  |
| MONA_28 | N |  |
| MONA_31 | P | SLC8A1-Ser298Phe (1.18%) |
| MONA_32 | N |  |
| MONA_34 | P | KPRP-Arg405Cys (0.41%) |
| MONA_35 | P | APC-Arg232* (0.43%), HCN1-Pro642Ser (2.29%),  HS3ST4-Arg359Gln (0.18%), KCTD8-Arg407Cys (0.37%) |
| MONA_36 | P | NYAP2-Arg453Gly (0.12%) |
| MONA_37 | N |  |
| MONA_39 | N |  |
| MONA_41 | P | LRFN5-Thr175Ile (0.25%) |

Table S3. Sequences of primers and probes

| Patient ID |  | Sequence (5’-3’) | Tm (°C) | GC-content (%) |
| --- | --- | --- | --- | --- |
| MONA_12 |  |  |  |  |
|  | Fwd | AAATCATCAGGAAGCAGAGCC | 57.9 | 47.6 |
|  | Rev | TGGGGAAGAGTGGGCTAGTG | 61.4 | 60.0 |
|  | Probe | FAM-CAGCTCCTGTTTGGCTCCCACCCTATGT-BHQ1 | 69.5 | 57.1 |
|  |  |  |  |  |
| MONA_17 |  |  |  |  |
|  | Fwd | CCTCCAGGCAGAGAGATAGGA | 61.8 | 57.1 |
|  | Rev | AGGGAGAAAACTCCCTCTAGTA | 58.4 | 45.5 |
|  | Probe | FAM-CCATCTTATTTCTAAGAAATCTGAAGATTCAAGGTGAAC-BHQ1 | 66.3 | 33.3 |
|  |  |  |  |  |
| MONA_18 |  |  |  |  |
|  | Fwd | CCTCCCAGGTTCAAGCGA | 58.2 | 61.1 |
|  | Rev | GCTCCTATTATCCTGTCCCTTTG | 60.6 | 47.8 |
|  | Probe | FAM-TGCAGGCAAAGGGATCTGCCGGTAGAA-BHQ1 | 68.0 | 55.6 |
|  |  |  |  |  |
| MONA_21 |  |  |  |  |
|  | Fwd | CAGTTTAGGCAAGCAGAGGTGA | 60.3 | 50.0 |
|  | Rev | AAGCCTCCCTGGATCTCCATA | 59.8 | 52.4 |
|  | Probe | FAM-CTAAGTATGATGGAAAGGTTCAGAGCTCAGG-BHQ1 | 66.8 | 45.2 |
|  |  |  |  |  |
| MONA_25 |  |  |  |  |
|  | Fwd | TCGGGAGACTATGAAATATTGTACT | 58.1 | 36.0 |
|  | Rev | CCTTGAAGCACTACACAGGC | 59.4 | 55.0 |
|  | Probe | FAM-AAGGTTCAGAGCTCAGGGGAGGATATGGAG-BHQ1 | 69.5 | 53.3 |

Table S4. EML4-ALK status of the blood samples from the Madsen cohort. P: Positive, N: Negative.

| Patient ID | Previous TKI |  | EML4-ALK status | |  | Reads spanning  breakpoint | | EML4 breakpoint position |
| --- | --- | --- | --- | --- | --- | --- | --- | --- |
|  |  |  | AVENIO | DNAfusion |  | AVENIO | DNAfusion |  |
| Madsen_1 | None |  | P | P |  | 115 | 51 | 42301001 |
| Madsen_2 | None |  | N | P |  | - | 2 | 42295516 |
| Madsen_3 | None |  | N | P |  | - | 2 | 42268786 |
| Madsen_4 | None |  | P | P |  | 775 | 491 | 42268753 |
| Madsen_5 | None |  | N | P |  | - | 3 | 42268334 |
| Madsen_6 | Crizotinib |  | N | P |  | - | 16 | 42276708 |
| Madsen_7 | Crizotinib |  | N | N |  | - | - | - |
| Madsen_8 | Crizotinib |  | N | P |  | - | 25 | 42299321 |
| Madsen_9 | Crizotinib |  | N | N |  | - | - | - |
| Madsen_10 | None |  | N | N |  | - | - | - |
| Madsen_11 | None |  | P | P |  | 275 | 153 | 42297153 |
| Madsen_12 | None |  | P | P |  | 72 | 30 | 42296711 |
| Madsen_13 | Crizotinib & Ceritinib |  | N | N |  | - | - | - |
| Madsen_14 | Crizotinib & Ceritinib |  | N | N |  | - | - | - |
| Madsen_15 | Crizotinib & Ceritinib |  | N | P |  | - | 6 | 42298968 |
| Madsen_16 | Crizotinib |  | N | N |  | - | - | - |
| Madsen_17 | None |  | N | P |  | - | 8 | 42325612 |
| Madsen_18 | Crizotinib |  | N | P |  | - | 2 | 42301190 |
| Madsen_19 | None |  | P | P |  | 45 | 31 | 42280719 |
| Madsen_20 | None |  | P | P |  | 25 | 16 | 42299509 |
| Madsen_21 | Crizotinib |  | N | N |  | - | - | - |
| Madsen_22 | None |  | P | P |  | 769 | 175 | 42279047 |
| Madsen_23 | None |  | N | N |  | - | - | - |
| Madsen_24 | None |  | N | N |  | - | - | - |
